# Social Representations of Healthcare Organisational Models and Professional Practice Among Public-Sector Physicians and Nurses in Portugal: A Prototypical Analysis

**DOI:** 10.3390/healthcare14162638

**Published:** 2026-08-20

**Authors:** Alexandre Fernandes, Gonçalo Santinha, Teresa Forte

**Affiliations:** 1 Governance, Competitiveness and Public Policies, Department of Social, Political and Territorial Sciences, University of Aveiro, 3810-193 Aveiro, Portugal; g.santinha@ua.pt; 2 Center for Research in Neuropsychology and Cognitive and Behavioral Intervention, University of Coimbra, 3000-115 Coimbra, Portugal; teresanforte@gmail.com

**Keywords:** social representations, prototypical analysis, healthcare professionals, public healthcare, private healthcare, public–private partnerships, professional practice

## Abstract

**Background/Objectives:** Healthcare workforce shortages and the challenges facing healthcare professionals are among the most pressing issues confronting contemporary health systems. This study examines the social representations of public, private, and public–private partnership (PPP) healthcare organisational models and professional practice elicited from public-sector physicians and nurses in Portugal. **Methods:** A cross-sectional survey was conducted between October 2022 and May 2023 using a non-probability sample of 308 physicians and nurses classified as public-sector professionals. Participants completed a free-association task concerning the public, private, and PPP healthcare models and their professional practice. The evoked terms were examined using prototypical analysis within the structural approach to Social Representations Theory. The representation of the public healthcare sector included elements such as accessibility, universality, equity, slowness, disorganisation, and resource limitations. The private healthcare sector was characterised by elements including cost, profit, speed, organisation, inaccessibility, and service quality. **Results:** The representation of PPPs included better management, efficiency, profit, interests, and quality, alongside a relatively frequent “No opinion” response. Professional practice was characterised by elements including caring, dedication, empathy, resilience, exhaustion, perceived undervaluation, and remuneration. **Conclusions:** These findings describe the social representations elicited from the present sample of public-sector physicians and nurses and should not be interpreted as evidence of objective differences between healthcare organisational models or as direct measures of motivation, resilience, burnout, sector preference, or working conditions. The findings are exploratory and hypothesis-generating and may inform future comparative research involving professionals working across different healthcare settings, as well as studies using dedicated measures of professional and occupational outcomes.

## 1. Introduction

Health systems are facing persistent and increasingly complex workforce challenges. Shortages of healthcare professionals, geographical imbalances in workforce distribution, and difficulties recruiting and retaining qualified staff can undermine the capacity of health systems to meet population needs, particularly during periods of increased demand or crisis [1,2].

Although these challenges pre-date the COVID-19 pandemic, the pandemic intensified many of them, exposing existing structural weaknesses and placing considerable physical, emotional, and organisational pressures on healthcare professionals [3,4,5]. It also reinforced the central importance of the healthcare workforce to the effective and sustainable functioning of health systems and highlighted the contribution of professionals’ knowledge, skills, and commitment to the delivery of care [6,7].

As health systems moved beyond the acute phase of the pandemic and confronted its longer-term consequences, workforce recruitment and retention became increasingly prominent policy concerns. Ensuring an adequate and sustainable healthcare workforce requires attention not only to the recruitment of new professionals but also to the organisational and employment conditions associated with retaining existing staff, particularly within public healthcare systems [8,9].

A substantial body of research has examined factors associated with healthcare workforce motivation, job satisfaction, and retention. This literature encompasses intrinsic and extrinsic motivational factors, working conditions, career development, work–life balance, and public service values [10,11]. Studies have identified remuneration, working conditions, difficulties in reconciling professional and personal life, and limited opportunities for career progression as factors that may affect healthcare professionals’ motivation and employment experiences [12,13,14]. These conditions have also been examined in relation to absenteeism, recruitment, and retention, including the challenges involved in attracting and retaining younger professionals [15,16].

Understanding healthcare professionals’ perspectives on their work and organisational environment therefore remains important. Social Representations Theory provides a framework for examining how groups construct and share meanings concerning socially relevant objects and how these meanings contribute to their understanding of social reality [17,18,19,20]. Social representations are not simple reflections of an objective reality; rather, they are socially constructed and dynamic systems of meaning that emerge through the interaction between individual experience, communication, and shared social contexts [21,22,23,24].

According to Abric [25], social representations fulfil four principal functions. The knowledge function enables individuals and groups to understand and interpret reality through forms of practical, socially shared knowledge. The identity function contributes to the construction and preservation of social identity and group distinctiveness. The guiding function provides a framework through which situations, behaviours, and practices may be interpreted and oriented. Finally, the justifying function enables individuals and groups to explain and legitimise positions, decisions, and conduct [26,27]. Together, these functions make Social Representations Theory particularly relevant to the study of how healthcare professionals make sense of their professional environment and the organisational contexts in which healthcare is provided.

Within healthcare, social representations can provide shared frameworks through which professionals interpret organisational contexts, professional roles, and everyday practice [28,29,30]. These socially constructed meanings may be related to attitudes, evaluations, and professional orientations, but they should be distinguished from individual-level psychological constructs. In particular, free-association data can identify salient meanings associated with professional practice and healthcare organisational models but do not constitute direct measures of intrinsic motivation, extrinsic motivation, public service motivation, burnout, or other psychological or occupational constructs. Accordingly, Social Representations Theory is used in the present study to examine how healthcare professionals construct shared meanings around different healthcare organisational models and their professional practice, rather than to measure professional motivation directly.

Research on healthcare professionals has frequently examined individual-level constructs such as motivation, job satisfaction, retention, working conditions, and public service values [31,32]. Although these approaches provide important evidence concerning healthcare workforce experiences and attitudes, they do not address the same question as a structural social-representations approach: how professionals spontaneously construct shared meanings around healthcare organisational models and which elements occupy more salient positions within those representations. This distinction is particularly relevant in Portugal, where public-sector physicians and nurses work within a health system characterised by the coexistence and interaction of public and private provision and by different organisational arrangements. To our knowledge, there is limited evidence on the structure and content of public-sector healthcare professionals’ social representations of public, private, and public–private partnership (PPP) healthcare models and of their own professional practice. The present study addresses this gap using free association and prototypical analysis to examine the salient and potentially central and peripheral elements of these representations.

Against this background, the present study aims to identify and characterise the social representations elicited from public-sector physicians and nurses in Portugal regarding different healthcare organisational models and their own professional practice. Using a free-association task and prototypical analysis, the study examines the salient elements and potential internal structure of these representations.

Accordingly, the study addresses the following research questions:

RQ1. What social representations do public-sector physicians and nurses evoke regarding public, private, and public–private partnership (PPP) healthcare organisational models?

RQ2. What social representations do public-sector physicians and nurses evoke regarding their professional practice?

## 2. Materials and Methods

### 2.1. The Portuguese Health System

The Portuguese health system is centred on the National Health Service (Serviço Nacional de Saúde, SNS), established according to a Beveridge-type model and providing universal coverage as a constitutional right. The SNS is predominantly financed through general taxation. Healthcare provision involves both public and private providers, with primary care physicians generally acting as gatekeepers for access to specialised and secondary care [33].

Alongside the SNS, healthcare provision in Portugal involves public, private, and social-sector organisations. The public sector plays the principal role in the provision and organisation of SNS services, while the social sector is particularly involved in long-term and continuing care, and private providers contribute substantially to areas such as diagnostic services and outpatient consultations [33]. The resulting system therefore combines predominantly public financing and provision with complementary participation from private and social-sector providers.

Over recent decades, several organisational and management reforms have sought to address pressures on service delivery, efficiency, and financial sustainability. These have included greater managerial autonomy for public hospitals, the use of public–private partnerships (PPPs) for hospital construction, financing, and operation, and contractual arrangements between public entities and private providers [34]. Such reforms have formed part of broader debates concerning the organisation, efficiency, sustainability, universality, and equity of healthcare provision.

More recently, reforms within the SNS have emphasised greater integration between different levels of care. Decree-Law No. 102/2023 of 7 November established Local Health Units (Unidades Locais de Saúde, ULS) as public business entities integrating primary and hospital care. The restructuring resulted in 39 ULSs and was intended to strengthen coordination across levels of care and respond to challenges associated with population ageing, increasing disease complexity, access, and the organisation of patient pathways within the SNS [35].

Healthcare workforce availability and distribution remain important challenges within the Portuguese health system. Shortages affect both historically underserved rural areas and some urban areas, with implications for geographical equity in access to care [36]. Absenteeism has also been identified as a workforce concern within the SNS. In 2023, healthcare professionals recorded approximately 4 million days of absence, representing a 24.9% increase compared with 2018 and an average increase of approximately 160,000 days of absence per year over this period [37].

Portugal has approximately 5.6 physicians and 7.4 nurses per 1000 inhabitants, although national averages conceal geographical disparities in workforce availability, particularly in rural areas [38]. Demographic changes are also evident within the SNS workforce. Women account for approximately 78% of SNS healthcare professionals, while more than half of the workforce is aged 45 years or older, compared with 42% in 2010 [37]. These characteristics provide relevant context for understanding current workforce distribution and sustainability challenges within the public healthcare system.

According to data from Statistics Portugal for 2022, Portugal had 243 hospitals, comprising 131 private hospitals, 111 public hospitals, and one hospital operating under a PPP arrangement [38]. The number of private hospitals had increased by 29 since 2010, illustrating changes in the composition of hospital provision over this period. However, sufficiently disaggregated data on the distribution of physicians and nurses across public and private employment are not available from the sources used here. This limits the possibility of determining whether the sectoral composition of the present sample reflects the distribution of physicians and nurses across healthcare sectors in Portugal. Accordingly, the composition of the analytical sample should be regarded as a characteristic of the present non-probability sample rather than as representative of the national sectoral distribution of healthcare professionals.

### 2.2. Participants

This study employed a cross-sectional survey design using non-probability sampling. Eligible participants were professionally active physicians and nurses working in Portugal. The questionnaire was disseminated electronically through public and private healthcare institutions across different Portuguese regions, which were asked to circulate the survey among eligible healthcare professionals. Participation was voluntary and anonymous.

Participants were also asked to report, in an open-text field, the healthcare institution or institutions in which they practised. Responses included spelling variants and abbreviations, different units belonging to the same hospital organisation and, in some cases, more than one institution per respondent. Consequently, although these data document a broad range of healthcare institutions represented in the sample, they do not provide a reliable count of the institutions that formally disseminated the questionnaire. Moreover, because the survey link was circulated institutionally, the total number of individual professionals who received the invitation could not be established; therefore, an individual response rate could not be calculated.

A total of 413 questionnaires were received. During data screening, 105 were excluded because the free-association component was incomplete or contained responses that could not be reliably interpreted for analysis. Exclusion was based on the completeness and interpretability of the free-association data rather than on participants’ employment sector. The final analytical sample therefore comprised 308 participants with valid free-association data.

The present study constitutes a secondary analysis of the free-association component of a broader survey that included additional measures and psychometric scales. Some variables collected through the broader survey have been examined in a previous publication [39]; however, the free-association data concerning the public, private and PPP healthcare models and professional practice analysed in the present study were neither examined nor reported in that publication. The present study therefore addresses a distinct research objective and applies a different analytical approach, grounded in Social Representations Theory and prototypical analysis, to examine the structure and content of participants’ social representations.

Given the non-probability sampling strategy, the final sample should not be interpreted as statistically representative of the Portuguese healthcare workforce, and no population-based margin of error is claimed. Sample adequacy was instead considered in relation to the requirements of prototypical analysis. Previous methodological literature indicates that substantially smaller samples may be sufficient to identify interpretable representational structures, whereas larger samples generally provide greater stability [38,40,41,42]. The final analytical sample of 308 participants was therefore considered adequate for the purposes of the present exploratory prototypical analysis.

Among the 308 participants, 81.49% were female, 40.58% were aged 41–50 years, 73.70% were nurses and 22.08% held management positions associated with their professional roles (Table 1). The analytical sample was therefore predominantly female and comprised a higher proportion of nurses (73.70%) than physicians (26.30%). These proportions describe the present non-probability sample and should not be interpreted as reproducing the professional or demographic distribution of the Portuguese public healthcare workforce. Sociodemographic and professional variables were collected to characterise the sample and contextualise the findings; no subgroup comparisons between physicians and nurses, between managerial and non-managerial professionals, or according to other sociodemographic characteristics were conducted in the present analysis.

All participants included in the analytical sample were classified as public-sector professionals according to the employment-sector variable used for sample characterisation. However, responses to the open-text workplace question indicated that some participants also reported activity in private healthcare organisations alongside their public-sector employment. The questionnaire did not include a dedicated measure of dual public–private practice, including its frequency, contractual characteristics, or the allocation of working time between sectors; consequently, dual practice could not be systematically quantified or analysed. Previous professional experience in private or PPP organisations was not assessed.

Although the questionnaire was disseminated through both public and private healthcare institutions, no participants classified exclusively as private-sector professionals were retained in the final analytical sample. Consequently, the study does not provide a direct comparison between professionals employed across different healthcare sectors. Rather, the representations of the public, private and PPP healthcare models reported here reflect the perspectives of a sample classified as public-sector professionals. The absence of participants classified exclusively as private-sector professionals may have introduced selection and non-response bias, as their representations may differ systematically from those captured in the present sample. Because this potential bias could not be statistically addressed in the absence of such respondents, the interpretation of the findings was restricted accordingly, and no generalisation to healthcare professionals across all organisational settings is intended.

### 2.3. Instrument

A questionnaire incorporating a free-association task was developed by the research team to explore participants’ social representations of different healthcare organisational models and their professional practice. The questionnaire was administered in Portuguese. Free association was selected because it enables the spontaneous elicitation of salient elements associated with a given social object and is therefore well suited to examining the content and potential structure of social representations [43].

The free-association component comprised four stimuli referring to the public healthcare sector, the private healthcare sector, public–private partnerships (PPPs), and participants’ profession and professional practice. The prompts were developed specifically for the broader survey by the research team and were not derived from a previously validated instrument. No definition or explanatory description of PPPs was provided as part of the free-association task, allowing participants to respond according to their own understanding of this organisational model.

The four stimuli were presented to all participants in the same fixed order. For each stimulus, participants were asked to provide three words or expressions that, in their opinion, best characterised the corresponding object. The instructions were as follows:

1. “Indicate three words or expressions that, in your opinion, best characterise the public healthcare sector.”
2. “Indicate three words or expressions that, in your opinion, best characterise the private healthcare sector.”
3. “Indicate three words or expressions that, in your opinion, best characterise public–private partnerships.”
4. “Indicate three words or expressions that, in your opinion, best characterise your profession and professional practice.”

For each stimulus, participants entered three evocations sequentially into separate response fields. The order of evocation used in the subsequent prototypical analysis corresponded directly to the sequence in which these responses were entered: the first response was assigned position 1, the second position 2, and the third position 3. These positions were subsequently used to calculate the mean order of evocation (MOE).

Before the main data collection, the questionnaire was pilot-tested with 10 participants to assess the clarity and comprehensibility of the instructions, questions, and response format. As pilot testing did not result in substantive changes to the free-association prompts or questionnaire structure, these participants were retained in the final analytical sample.

In addition to the free-association task, the broader questionnaire collected sociodemographic and professional information, including sex, age group, profession, educational level, management responsibilities, employment sector, geographical region, and current healthcare institution(s). Employment sector was recorded using predefined response categories, whereas current healthcare institution(s) were reported in an open-text field. These variables were used in the present study to characterise the analytical sample and contextualise the findings. No subgroup comparisons between physicians and nurses, between managerial and non-managerial professionals, or according to other sociodemographic characteristics were conducted in the present analysis.

### 2.4. Procedure and Data Analysis

Prototypical analysis, developed by Vergès [40], is a widely used approach for examining the structure of social representations using free-association data. The technique considers two complementary indicators: the frequency with which an element is evoked and its mean order of evocation (MOE). Taken together, these indicators allow evoked elements to be organised according to their relative salience and potential structural position within a social representation [40,44].

Prototypical analysis is grounded in Central Core Theory, which proposes that a social representation is organised around central and peripheral systems. The central core comprises a limited number of elements that organise and give meaning to the representation and are generally characterised by greater stability and consensus within a social group. By contrast, the peripheral system is more flexible and context-sensitive, allowing the representation to accommodate individual experiences and specific situations while contributing to the maintenance of the central core [20,41,44]. Prototypical analysis identifies elements that are potentially central on the basis of their frequency and order of evocation; confirmation of structural centrality requires complementary methods specifically designed to test centrality.

The prototypical analysis organised the evoked elements into four quadrants according to their frequency and MOE:

- Central Core (Q1): high frequency and early evocation, comprising the most salient and potentially central elements of the representation;
- First Periphery (Q2): high frequency and late evocation, comprising widely shared elements that are evoked later;
- Contrast Zone (Q3): low frequency and early evocation, comprising salient elements that may reflect representations held by smaller subgroups;
- Second Periphery (Q4): low frequency and late evocation, comprising less frequent and less salient peripheral elements.

The frequency and MOE thresholds were defined with reference to established methodological recommendations for prototypical analysis [38,40,41,42,44,45]. Because three sequential evocations were requested for each stimulus, the midpoint of the possible positions (1, 2, and 3) provided a MOE threshold of 2. Elements with a MOE < 2 were therefore classified as early evocations, whereas those with a MOE ≥ 2 were classified as later evocations. Methodological approaches to defining the frequency threshold vary and may be based on the distribution of observed frequencies or on a predefined proportion of the sample [44,45]. In the present study, a frequency cut-off of 15 was adopted: elements with f > 15 were classified as high-frequency elements, whereas those with f ≤ 15 were classified as low-frequency elements. No formal sensitivity analysis using alternative frequency or MOE thresholds was conducted; consequently, the quadrant allocation of elements close to these cut-offs should be interpreted with caution.

For tabular presentation, a minimum frequency of 7 was adopted for low-frequency elements in the Contrast Zone and Second Periphery. Accordingly, while f > 15 determined the distinction between high- and low-frequency elements for quadrant allocation, f ≥ 7 represented the minimum frequency considered for reporting low-frequency elements in the summary tables. This presentation criterion did not affect the calculation of frequencies or MOE, or the allocation of elements within the prototypical analysis. Elements below this minimum frequency remained part of the analytical dataset but were not displayed in the tables.

Before prototypical analysis, the Portuguese-language free-text responses underwent manual lexical and semantic standardisation. Obvious spelling variants were corrected, singular and plural forms referring to the same concept were harmonised, and synonymous words or expressions were consolidated when they conveyed the same meaning. Multiword expressions were retained or grouped where necessary to preserve the intended concept, and semantically similar responses were combined only when they represented the same underlying meaning. Coding decisions were discussed among the researchers and resolved by consensus, with the aim of reducing lexical dispersion while preserving participants’ intended meanings. Coding was not conducted as an independent double-coding procedure, and no formal inter-rater reliability statistic, such as Cohen’s kappa, was calculated. No formal coding dictionary or independent audit trail of individual grouping decisions was maintained during the original preprocessing procedure. These features may limit the reproducibility of the preprocessing stage. Standardisation and categorisation were conducted in Portuguese, and the resulting terms and categories were subsequently translated into English for reporting.

Following preprocessing, all valid standardised evocations were entered into OpenEvoc (version 1.1—Montevideo) for prototypical analysis. The software was used to calculate the frequency and MOE of each standardised element and to classify elements into the four quadrants according to the predefined thresholds. The analysis was conducted separately for each of the four stimuli: public healthcare sector, private healthcare sector, PPPs, and professional practice. Physicians and nurses were analysed jointly because the study focused on social representations at the level of the analytical sample of public-sector healthcare professionals rather than on profession-specific representational structures. Both professional groups were therefore treated as members of the broader group addressed by the research questions. This analytical approach does not imply that physicians and nurses necessarily share identical representational structures.

The final analytical dataset contained three valid evocations for each of the 308 participants for each of the four stimuli, resulting in 924 evocations per stimulus and 3696 evocations overall. No missing evocation fields remained in the final analytical dataset because questionnaires with incomplete or uninterpretable free-association responses had been excluded during data screening. Before lexical and semantic standardisation, 445 distinct forms were identified for the public healthcare sector, 523 for the private healthcare sector, 588 for PPPs, and 472 for professional practice. Following standardisation, these were consolidated into 333, 431, 469, and 386 distinct terms or semantic categories, respectively. All valid standardised evocations contributed to the calculation of frequency and MOE. The elements displayed in the four-quadrant tables accounted for 506 of the 924 public-sector evocations (54.8%), 362 of the 924 private-sector evocations (39.2%), 330 of the 924 PPP evocations (35.7%), and 436 of the 924 professional-practice evocations (47.2%). The remaining evocations comprised lower-frequency elements that contributed to the overall analysis but were not displayed in the summary tables.

## 3. Results

Prototypical analysis identified salient and potentially central and peripheral elements of participants’ social representations of the public healthcare sector, private healthcare sector, public–private partnerships (PPPs), and professional practice. Based on frequency and mean order of evocation (MOE), the standardised terms were organised into four quadrants: Central Core (Q1), First Periphery (Q2), Contrast Zone (Q3), and Second Periphery (Q4).

The final analytical dataset comprised 3696 valid evocations, corresponding to 924 evocations for each of the four stimuli. No missing evocation fields remained in the final analytical sample. Before lexical and semantic standardisation, 445 distinct forms were identified for the public healthcare sector, 523 for the private healthcare sector, 588 for PPPs, and 472 for professional practice. Following standardisation, these were consolidated into 333, 431, 469, and 386 distinct terms or semantic categories, respectively. The elements displayed in the four-quadrant tables accounted for 506 evocations (54.8%) for the public healthcare sector, 362 (39.2%) for the private healthcare sector, 330 (35.7%) for PPPs, and 436 (47.2%) for professional practice.

Regarding the public healthcare sector (Table 2), the Central Core (Q1) included “Slow” (f = 52; MOE = 1.69), “Disorganised” (f = 49; MOE = 1.78), “Accessibility” (f = 44; MOE = 1.68), “Free” (f = 33; MOE = 1.97), “Universal” (f = 31; MOE = 1.39), “Equity” (f = 28; MOE = 1.64), “Decadent” (f = 18; MOE = 1.39), “Equality” (f = 18; MOE = 1.89), and “Dedication” (f = 16; MOE = 1.69). The First Periphery (Q2) included “Lack of human and material resources” (f = 30; MOE = 2.27), “Quality” (f = 21; MOE = 2.43), “Low salary” (f = 19; MOE = 2.11), and “Safety” (f = 16; MOE = 2.19). Lower-frequency elements were distributed across the Contrast Zone (Q3) and Second Periphery (Q4), as shown in Table 2.

Regarding the private healthcare sector (Table 3), the Central Core (Q1) comprised “Expensive” (f = 63; MOE = 1.52), “Profit” (f = 56; MOE = 1.64), “Speed” (f = 55; MOE = 1.87), “Organised” (f = 28; MOE = 1.82), and “Inaccessibility” (f = 26; MOE = 1.88). “Service quality” (f = 51; MOE = 2.04) was located in the First Periphery (Q2). Lower-frequency elements included “Business”, “Accessibility”, “Efficiency”, “Innovative”, and “High salary” in the Contrast Zone (Q3), and “Availability”, “No opinion”, and “Elitist” in the Second Periphery (Q4).

Regarding PPPs (Table 4), the Central Core (Q1) comprised “Better management” (f = 46; MOE = 1.78), “Efficiency” (f = 45; MOE = 1.69), “Profit” (f = 37; MOE = 1.70), “Interests” (f = 23; MOE = 1.91), “Quality” (f = 22; MOE = 1.82), and “Organised” (f = 20; MOE = 1.80). The First Periphery (Q2) included “No opinion” (f = 45; MOE = 2.16), “Accessibility” (f = 24; MOE = 2.13), and “Better conditions” (f = 19; MOE = 2.16). Lower-frequency elements were distributed between the Contrast Zone (Q3) and Second Periphery (Q4), as shown in Table 4.

“No opinion” (f = 45; MOE = 2.16) was retained in the analysis because it was explicitly entered by participants in response to the PPP stimulus rather than treated as missing data. As it does not describe a substantive attribute of PPPs, however, it should be distinguished from the semantic content of the other evocations. Its frequency indicates that a substantial subset of participants did not provide a substantive characterisation of the PPP model; the reasons for these responses cannot be determined from the free-association data alone.

Regarding professional practice (Table 5), the Central Core (Q1) comprised “Caring” (f = 86; MOE = 1.66), “Dedication” (f = 51; MOE = 1.98), “Empathy” (f = 44; MOE = 1.73), “Resilience” (f = 36; MOE = 1.83), “Exhausting” (f = 23; MOE = 1.57), and “Altruism” (f = 22; MOE = 1.95). The First Periphery (Q2) comprised “Undervalued” (f = 69; MOE = 2.28), “Humanism” (f = 23; MOE = 2.04), and “Low salary” (f = 23; MOE = 2.22). “Professionalism” and “Competence” were located in the Contrast Zone (Q3), whereas “Responsibility”, “Challenging”, “Compassion”, and “Knowledge” were located in the Second Periphery (Q4).

## 4. Discussion

The present study explored the social representations elicited from public-sector physicians and nurses in Portugal regarding public, private, and public–private partnership (PPP) healthcare organisational models and their own professional practice. Prototypical analysis identified distinct representational configurations across the four stimuli. These findings concern meanings spontaneously elicited from professionals classified as working in the public sector and should not be interpreted as direct evidence of objective differences in working conditions, organisational performance, professional motivation, or employment experiences across healthcare sectors.

Data collection took place during a period in which healthcare professionals and organisations continued to experience pressures associated with the COVID-19 pandemic. This context should be considered when interpreting the salience of the representations identified. Previous research has documented substantial organisational and occupational pressures experienced by healthcare workers during and following the pandemic [46,47,48,49]. However, the present free-association data do not allow the extent to which this context influenced the observed representations to be determined.

### 4.1. Representations of Public, Private, and Public–Private Organisational Models

The representation of the public healthcare sector was characterised by the coexistence of organisationally critical and value-oriented elements. “Slow” and “Disorganised” occupied salient positions alongside “Accessibility”, “Free”, “Universal”, “Equity”, and “Equality”. Rather than supporting an overall positive or negative characterisation of the public sector, this configuration suggests that participants’ representation encompassed different dimensions of the public healthcare model, including perceived organisational constraints and principles traditionally associated with universal healthcare provision.

A notable contrast was observed between “Slow” in the representation of the public sector and “Speed” in that of the private sector. “Expensive”, “Profit”, “Organised”, and “Inaccessibility” were also salient in participants’ representations of private healthcare. From the perspective of the public-sector professionals included in this study, these patterns suggest that the two organisational models were differentiated through attributes relating to responsiveness, organisation, cost, and accessibility.

This contrast may be considered in relation to the literature on dual practice in healthcare. Healthcare professionals in Portugal may combine public and private professional activity, and experience across different organisational environments may contribute to how these sectors are represented [50,51,52,53,54,55,56,57]. Accordingly, the opposition between “Slow” and “Speed” may contribute to understanding how participants differentiated the two models. Nevertheless, the present study did not systematically assess dual practice, employment trajectories, sector preferences, or the reasons underlying employment choices. The findings therefore cannot establish whether these representations influence decisions to work in, leave, or combine different healthcare sectors. Any such relationship remains to be examined using dedicated measures and appropriate study designs.

The representation of PPPs also encompassed different organisational dimensions. “Better management”, “Efficiency”, “Profit”, “Interests”, “Quality”, and “Organised” were among the salient elements. The presence of “Better management” and “Efficiency” can be considered alongside evidence from the Portuguese healthcare context suggesting that PPP hospital models may achieve favourable economic and operational outcomes while maintaining service quality [58]. This comparison should, however, be interpreted cautiously, as the present findings concern participants’ representations of PPPs rather than an objective assessment of PPP performance.

The simultaneous presence of “Profit” and “Interests” indicates that economic and interest-related considerations formed part of participants’ representation of PPPs. These elements may be considered in relation to broader questions of governance, accountability, and the relationship between public-service objectives and private-sector participation. However, the free-association design does not establish how participants understood these terms or whether they attached positive or negative evaluations to them.

An additional finding was the relatively high frequency of “No opinion” in response to the PPP stimulus. Unlike the other terms, “No opinion” does not constitute a substantive attribute of the organisational model. Its frequency may reflect uncertainty, limited familiarity, or the absence of a consolidated representation of PPPs among some participants. However, because participants were not subsequently asked to explain their responses, the reasons for these responses cannot be determined from the present data. Future qualitative research could examine how healthcare professionals understand PPP arrangements and whether familiarity with these models is related to the content and structure of their representations.

All these representations were elicited from professionals classified as working in the public sector. The observed contrasts between public, private, and PPP healthcare should therefore not be interpreted as demonstrating actual differences in working conditions or organisational performance across these settings. Participants’ representations may have been shaped by several sources, including their own professional experience, dual practice in some cases, interactions with colleagues, previous employment experiences, and broader professional or societal discourses. The present design does not allow the relative contribution of these possible sources to be determined.

### 4.2. Representations of Professional Practice

The representation of professional practice included “Caring”, “Dedication”, “Empathy”, “Resilience”, “Exhausting”, and “Altruism” in the Central Core quadrant, while “Undervalued”, “Humanism”, and “Low salary” appeared in the First Periphery. This configuration suggests the coexistence of meanings relating to interpersonal and value-oriented aspects of healthcare work with elements concerning perceived demands, professional recognition, and remuneration.

The presence of “Caring”, “Empathy”, “Dedication”, and “Altruism” is consistent with literature emphasising prosocial and service-oriented values in healthcare and public-sector employment [59,60]. Nevertheless, these evocations should not be interpreted as direct measures of intrinsic motivation, altruistic motivation, or public service motivation. Rather, they indicate that these meanings formed part of participants’ spontaneous representations of their professional practice.

Similarly, “Resilience” emerged as a salient evoked term, but its presence does not demonstrate participants’ psychological resilience. The same caution applies to “Exhausting”, “Undervalued”, and “Low salary”. These terms indicate that strain, recognition, and remuneration formed part of participants’ representation of professional practice. They may be considered alongside previous literature documenting burnout, occupational stress, and workforce pressures among healthcare professionals, particularly during and following the COVID-19 pandemic [39,61,62]. However, burnout, psychological resilience, job satisfaction, and occupational stress were not directly measured in the present analysis, and no conclusions about their prevalence or severity can therefore be drawn.

The present findings likewise cannot demonstrate that the identified representations determine retention, workforce migration, career choices, or work–life balance. Previous research provides grounds for considering these constructs when examining healthcare workforce dynamics but establishing relationships between social representations and employment behaviour would require studies combining free-association approaches with validated measures of motivation, occupational well-being, employment intentions, and longitudinal career outcomes.

### 4.3. Prototypical Analysis and Social Representations

Prototypical analysis provided a structured means of examining the organisation of meanings spontaneously associated with the four stimuli. Grounded in Central Core Theory, this approach considers both frequency and mean order of evocation (MOE), allowing potentially central elements to be distinguished from more peripheral elements of a representation [63,64,65]. The location of an element in the Central Core quadrant should nevertheless be interpreted as an indication of potential centrality rather than definitive confirmation of its structural status, which would require complementary centrality-testing procedures [63,65].

The present findings can also be considered alongside previous research applying structural approaches to social representations among healthcare professionals. Coelho et al. [66], for example, used structural analysis to examine healthcare professionals’ social representations of COVID-19. Although the representational object and context differ from those examined here, their study illustrates the application of structural approaches to the organisation of shared meanings among healthcare professionals. The present study applies this methodological perspective to public-sector physicians’ and nurses’ representations of healthcare organisational models and professional practice in Portugal.

From a structural perspective, the coexistence of apparently contrasting elements within the same representation is noteworthy. The representation of the public healthcare sector, for example, incorporated “Accessibility”, “Universal”, “Equity”, and “Equality” alongside “Slow” and “Disorganised”. Similarly, the private-sector representation combined “Speed” and “Organised” with “Expensive”, “Profit”, and “Inaccessibility”. These configurations illustrate why the representations identified here should not be reduced to uniformly positive or negative evaluations.

Nevertheless, the present analysis is based on frequency and order of evocation and does not independently establish the meaning participants attributed to each term. Individual evocations should therefore not be treated as direct measures of psychological constructs or attitudes. Similarly, elements located in the Central Core quadrant are regarded as potentially central rather than as definitively confirmed components of the structural core [63,65].

### 4.4. Implications

The findings provide exploratory evidence of how public-sector physicians and nurses represented different healthcare organisational models and their professional practice. Within the public-sector representation, the salience of “Slow”, “Disorganised”, and “Lack of human and material resources” identifies administrative processes, organisational functioning, and resource availability as relevant dimensions of participants’ perceptions of the SNS. At the same time, “Accessibility”, “Universal”, “Equity”, and “Equality” indicate the salience of principles associated with universal public healthcare provision.

These findings may help identify areas warranting further investigation rather than serving as direct evidence of organisational deficiencies. Future organisational assessments could, for example, examine whether perceived administrative slowness corresponds to measurable workflow inefficiencies, whether perceived resource constraints vary across institutions, and how professionals’ perceptions relate to objective indicators of staffing and service delivery.

The representation of professional practice similarly identifies professional recognition and remuneration as areas that may warrant further investigation alongside the interpersonal and value-oriented dimensions of healthcare work. Organisational approaches to recognition, professional development, supportive management, and staff participation could be examined in future research. However, the appropriateness or effectiveness of specific interventions cannot be inferred from the present free-association findings and would require direct evaluation.

### 4.5. Limitations

This study has several limitations. First, the use of non-probability sampling and voluntary participation limits the statistical generalisability of the findings and introduces the possibility of self-selection bias. An individual response rate could not be calculated because the questionnaire was disseminated institutionally and the number of professionals who received the invitation was unknown. Of the 413 questionnaires received, 105 were excluded because the free-association component was incomplete or could not be reliably interpreted; the original screening records did not allow these exclusions to be retrospectively classified in greater detail. As participation was anonymous, potential duplicate submissions could not be definitively identified. Moreover, although the questionnaire was disseminated through both public and private healthcare institutions, the final analytical sample comprised professionals classified as working in the public sector, some of whom also reported private-sector activity. Dual practice and previous sector experience were not systematically assessed. The findings concerning private healthcare and PPPs therefore reflect the representations of public-sector professionals and cannot be generalised to professionals working across different organisational settings.

Second, physicians and nurses were analysed jointly, although their distinct occupational identities, professional roles, and work experiences may be associated with different representational structures, particularly in relation to professional practice. The pooled analysis may therefore have obscured profession-specific patterns. The sample was also predominantly composed of nurses and women, and no subgroup analyses were conducted according to profession, management status, or other sociodemographic characteristics. Potential institutional clustering could not be formally assessed. Data collection also took place during a period of continuing pressures associated with the COVID-19 pandemic, which may have influenced the salience of particular evocations. In addition, the four stimuli were presented in a fixed order, and potential order or carry-over effects cannot therefore be excluded. Lexical and semantic standardisation involved interpretive decisions reached through researcher consensus, without independent double-coding, formal inter-rater reliability assessment, a formal coding dictionary, or an independent audit trail. Standardisation was conducted in Portuguese, and the resulting terms were subsequently translated into English for reporting, with the possibility of some loss of semantic nuance.

Finally, prototypical analysis identifies potentially central elements based on frequency and mean order of evocation, but structural centrality was not independently confirmed through complementary testing. No sensitivity analysis using alternative thresholds was conducted, and elements close to the adopted cut-offs should therefore be interpreted cautiously. The free-association task also did not directly measure motivation, burnout, psychological resilience, job satisfaction, work–life balance, sector preference, retention intentions, workforce migration, or objective working conditions. Relationships between the representations identified here and these constructs should therefore be regarded as hypothesis-generating and require investigation using dedicated and validated measures.

## 5. Conclusions

This study provides insight into the social representations elicited from public-sector physicians and nurses in Portugal regarding public, private, and public–private partnership (PPP) healthcare organisational models and their own professional practice. Through free association and prototypical analysis, distinct configurations of salient and potentially central and peripheral elements were identified across the four representational objects examined.

Participants’ representation of the public healthcare sector combined principles associated with universal healthcare provision, including accessibility, universality, equity, and equality, with elements relating to slowness, disorganisation, and resource constraints. The private healthcare sector was represented through elements including speed, organisation, service quality, cost, profit, and inaccessibility, while PPPs were associated with better management, efficiency, quality, profit, and interests. The relatively frequent “No opinion” response to the PPP stimulus also suggests that some participants did not provide a substantive characterisation of this organisational model. These findings reflect the representations of the present sample of public-sector professionals and should not be interpreted as evidence of objective differences in organisational performance or working conditions across healthcare settings.

Professional practice was represented through elements including caring, dedication, empathy, resilience, altruism, exhaustion, perceived undervaluation, and remuneration. These evocations provide insight into the meanings participants associated with their professional practice but do not constitute direct measures of motivation, psychological resilience, burnout, job satisfaction, work–life balance, sector preference, retention intentions, or workforce mobility.

Free association and prototypical analysis provided an exploratory approach to examining the organisation and relative salience of these shared meanings. Elements located in the Central Core quadrant should be regarded as potentially central rather than as independently confirmed components of the representational core. Accordingly, the findings are exploratory and hypothesis-generating rather than evidence of stable structural relationships or causal mechanisms.

From a practical perspective, perceived administrative responsiveness, resource availability, professional recognition, and remuneration emerge as dimensions that may warrant further investigation. These implications are preliminary and require complementary evidence before informing organisational or policy interventions. Future research should include professionals working across public, private, and PPP settings and systematically assess dual practice and previous sector experience. Comparative analyses across professional groups and longitudinal research could further examine variation and change in these representations. Combining social-representation approaches with validated measures of motivation, burnout, resilience, job satisfaction, work–life balance, sector preference, and retention intentions would also help determine whether and how the representational patterns identified here relate to professional attitudes, employment experiences, and behaviour.

## Figures and Tables

**Table 1:** Sample characteristics.

Characteristics	N
**Sex**	Female: 251 (81.49%) Male: 57 (18.51%)
**Age group**	≤30 years old: 0 (0.00%) 31–40 years old: 89 (28.90%) 41–50 years old: 125 (40.58%) 51–60 years old: 66 (21.43%) Over 60 years old: 28 (9.09%)
**Profession**	Nurse: 227 (73.70%) Physician: 81 (26.30%)
**Educational level**	Degree: 128 (41.56%) Master: 106 (34.42%) Post grad: 66 (21.43%) PhD: 8 (2.60%)
**Management**	Yes: 68 (22.08%) No: 240 (77.92%)
**Work sector**	Sector classification: Public sector—100%

**Table 2:** Evoked terms in the public healthcare sector.

		Mean Order of Evocation (Public Sector)					
		**<2** **Central Core (Q1)**			**≥2** **First Periphery (Q2)**		
		Term evoked	f	MOE	Term evoked	f	MOE
Mean Frequency	Higher Frequency (f > 15)	Slow	52	1.69	Lack of Human and Material Resources	30	2.27
		Disorganised	49	1.78	Quality	21	2.43
		Accessibility	44	1.68	Low Salary	19	2.11
		Free	33	1.97	Safety	16	2.19
		Universal	31	1.39			
		Equity	28	1.64			
		Decadent	18	1.39			
		Equality	18	1.89			
		Dedication	16	1.69			
		**Contrast Zone (Q3)**			**Second Periphery (Q4)**		
Mean Frequency	Lower Frequency (f ≤ 15)	Competence	14	1.64	Caring	15	2.60
		Trust	9	1.56	Disinvestment	14	2.14
		Exhaustion	7	1.86	Mismanagement	14	2.29
					Demotivation	13	2.23
					Devaluation	13	2.31
					Bureaucratic	12	2.08
					Professionalism	10	2.20
					Resilience	10	2.20

**Table 3:** Evoked terms in the private healthcare sector.

		Mean Order of Evocation (Private Sector)					
		**<2** **Central Core (Q1)**			**≥2** **First Periphery (Q2)**		
		Term evoked	f	MOE	Term evoked	f	MOE
Mean Frequency	Higher Frequency (f > 15)	Expensive	63	1.52	Service Quality	51	2.04
		Profit	56	1.64			
		Speed	55	1.87			
		Organised	28	1.82			
		Inaccessibility	26	1.88			
		**Contrast Zone (Q3)**			**Second Periphery (Q4)**		
Mean Frequency	Lower Frequency (f ≤ 15)	Business	15	1.53	Availability	9	2.11
		Accessibility	11	1.73	No opinion	9	2.22
		Efficiency	11	1.73	Elitist	8	2.00
		Innovative	10	1.50			
		High Salary	10	1.90			

**Table 4:** Evoked terms in public–private partnerships.

		Mean Order of Evocation (Public–Private Partnerships)					
		**<2** **Central Core (Q1)**			**≥2** **First Periphery (Q2)**		
		Term evoked	f	MOE	Term evoked	f	MOE
Mean Frequency	Higher Frequency (f > 15)	Better Management	46	1.78	No opinion	45	2.16
		Efficiency	45	1.69	Accessibility	24	2.13
		Profit	37	1.70	Better conditions	19	2.16
		Interests	23	1.91			
		Quality	22	1.82			
		Organised	20	1.80			
		**Contrast Zone (Q3)**			**Second Periphery (Q4)**		
Mean Frequency	Lower Frequency (f ≤ 15)	Quickness	12	1.92	Security	10	2.30
		Disorganised	11	1.45	Difficult Accessibility	8	2.00
					High Salary	8	2.00

**Table 5:** Evoked terms related to professional practice.

		Mean Order of Evocation (Professional Practice)					
		**<2** **Central Core (Q1)**			**≥2** **First Periphery (Q2)**		
		Term evoked	f	MOE	Term evoked	f	MOE
Mean Frequency	Higher Frequency (f > 15)	Caring	86	1.66	Undervalued	69	2.28
		Dedication	51	1.98	Humanism	23	2.04
		Empathy	44	1.73	Low Salary	23	2.22
		Resilience	36	1.83			
		Exhausting	23	1.57			
		Altruism	22	1.95			
		**Contrast Zone (Q3)**			**Second Periphery (Q4)**		
Mean Frequency	Lower Frequency (f ≤ 15)	Professionalism	9	1.44	Responsibility	15	2.07
		Competence	8	1.63	Challenging	11	2.00
					Compassion	8	2.00
					Knowledge	8	2.38

## Data Availability

The data are not publicly available due to privacy or ethical restrictions.
